# Construction of a High-Density Genetic Map and Its Application for QTL Mapping of Leaflet Shapes in Mung Bean (*Vigna radiata* L.)

**DOI:** 10.3389/fgene.2020.01032

**Published:** 2020-09-30

**Authors:** Jie Wang, Jianling Li, Zhenxing Liu, Xingxing Yuan, Suhua Wang, Honglin Chen, Xin Chen, Xuzhen Cheng, Lixia Wang

**Affiliations:** ^1^Institute of Crop Sciences, Chinese Academy of Agricultural Sciences, Beijing, China; ^2^College of Life Science, Yangtze University, Jingzhou, China; ^3^Tangshan Academy of Agricultural Sciences, Tangshan, China; ^4^Institute of Industrial Crops, Jiangsu Academy of Agricultural Sciences, Nanjing, China

**Keywords:** *Vigna radiata*, high-density map, leaflet shape, QTL, SNP

## Abstract

Mung bean (*Vigna radiata* L.) is an important but understudied food legume in Asia and now worldwide. Genetic studies may help to accelerate the exploitation of new genes for breeding in this crop. Here, we used a recombination inbred line population to construct an SNP genetic linkage map by genome sequencing technology. We obtained 21,508 high-quality SNP markers integrated into 1,946 bin markers that were mapped onto 11 linkage groups (LGs) with 99–258 bin markers per LG. The total genetic length of the map was 1060.2 cM (38.76–168.03 cM per LG), with an average distance between markers of 0.54 cM. However, there were 18 gaps >5 cM, distribution on LG1, 3, 5, 7, and 9. Gene mapping for lobed and indented leaflets was conducted using the map. A major quantitative trait locus (QTL) associated with indented leaflets was detected on chromosome 10, with phenotypic variation explained (PVE) values of 39.7% and 45.4% under two different environments. Several QTLs for lobed leaflets were detected and most of them were tightly linked together on Chromosome 3. However, only one major QTL, which explained the largest phenotypic variation (27.7–69.5%), was stably detected under two different environments using both R and Q methods. In the two main stable QTLs regions on chromosomes 3 and 10, candidate genes for regulating the molecular mechanism of different leaflet shapes were detected by functional annotation. The overlap of major QTLs under different environments indicated that the present map would be good enough for precisely mapping genes, and both the QTL analysis and gene prediction were useful for investigating the mechanism of leaf development in mung bean or legumes.

## Introduction

Mung bean (*Vigna radiata* L.), a cultivated species in genus *Vigna* (2n = 2 × = 22), has a genome size of about 500 Mb (Yang et al., 2014). It is an important plant protein resource and a potentially healthy food (Nair et al., 2013; Shi et al., 2016) that has traditionally grown across Asia (Tomooka et al., 2002). Mung bean production has spread to most parts of the world in recent decades. The worldwide demand of mung bean has greatly increased because of its healthy properties, especially the identification of physiological components in the seeds (Yao et al., 2013), that have attracted much interest. Therefore, breeding new varieties with high yield and high quality is urgently required. Genetic and genomic study can help to improve the efficiency of mining for new genes and breeding of important varieties, but mung bean has been less studied than other major crops.

Linkage map construction and gene mapping are important for genetic and genomic studies. A number of genetic linkage maps for mung bean, based mainly on restriction fragment length polymorphism (RFLPs) (Young et al., 1992; Chaitieng et al., 2002; Humphry et al., 2002) or simple sequence repeats (SSRs) (Kajonphol et al., 2012; Sompong et al., 2012; Wang et al., 2016; Singh et al., 2018), have been reported previously. However, most of these maps were low density because of the limited number of polymorphic markers, although primarily mappings of genes associated with bruchid resistance (Chen et al., 2012; Wang et al., 2016), domesticated traits (Isemura et al., 2012), and agronomic traits (Kajonphol et al., 2012) were reported. With the release of the whole genome sequence of mung bean (Yang et al., 2014), rapid progress on gene tagging and candidate genes associated with resistance to pests such as seed beetles (*Coleoptera: Bruchidae*) and disease also has been made (Chotechung et al., 2016; Kaewwongwal et al., 2017; Mathivathana et al., 2019). Single nucleotide polymorphism (SNP) maps, essential for precise gene mapping, have been constructed for species related to mung bean, such as black gram and wild cowpea for bruchid resistance (Amkul et al., 2019; Somta et al., 2019), cowpea for yield traits (Pan et al., 2017), and adzuki bean for flowering time (Liu et al., 2016). However, similar studies are rare in mung bean. To enhance the genetic study of mung bean, we constructed an SNP map using a recombinant inbred line (RIL) population. The original parents of the RILs were both cultivated genotypes with different major agronomic traits. To validate the usefulness of the SNP map, we conducted a quantitative trait locus (QTL) analysis of two rare leaflet shapes of mung bean and predicted the candidate genes associated with these traits. The results will help to accelerate the genetic research of mung bean and contribute to the breeding of improved varieties.

## Materials and Methods

### Plant Material

*Dahuaye*, green seeded, is a landrace with lobed leaflet, and *Jilyu 9-1*, black-seeded, is a natural mutant with indented leaflet that was obtained from field tests, and the indented leaflet was stably inherit after several season’s propagation. A cross between these two genotypes in 2012 in Beijing produced a hybrid that was sown in the following season. After harvest of the F_1_ plant, each line of the F_2_ generation was planted continually for several years by the single seed descend method, until an RIL_8_ population was developed in 2017 for high-density map construction.

### DNA Preparation, Library Construction, and Sequencing

The RIL population was continually planted in 2018 and 2019 with 10 seeds for each line. Young leaves of 10 plants from each of 190 randomly selected lines were collected at the 2019 season. DNA was extracted using the CTAB method (Doyle and Doyle, 1987). The quality of the extracted DNA was checked using a NanoPhotometer^®^ spectrophotometer (IMPLEN, Westlake Village, CA, United States) and the concentration was determined using a Qubit^®^ DNA Assay Kit and a Qubit^®^ 2.0 Fluorometer (Life Technologies, Carlsbad, CA, United States).

A total of 1.5 μg genomic DNA per sample was fragmented by sonication to 350 bp, then end polished, A-tailed, and ligated with the full-length adapter. After PCR amplification, product purification (AMPure XP system, Beckman Coulter, Beverly, MA, United States), and size distribution assessment using an Agilent 2100 Bioanalyzer, the target fragments were quantified by real-time PCR. The prepared libraries were sequenced on an Illumina NovaSeq 6000 PE150 platform (Illumina, San Diego, CA, United States) and 150-bp paired-end reads were generated with insert sizes of about 350 bp.

Raw reads in *fasta* format were processed using in-house C scripts (Biomarker Technologies, Beijing, China) for quality control (QC) to ensure the reliability and that the reads were free of base-calling duplicates, adapter contamination, and other artificial biases. The QC process removed reads with ≥10% unidentified nucleotides, reads with >50% low-quality bases, reads with >10 nucleotides aligned to the adapter allowing ≤10% mismatches, and putative PCR duplicates generated by PCR amplification in the library construction process.

### Mapping to the Reference Genome and SNPs Detection

The genome sequence of mung bean (GenBank: GCA_000741045.2) was used as the reference genome (Yang et al., 2014). The clean reads in each library were aligned against the reference genome (settings: mem -t 4 -k 32 –M-R) using the Burrows-Wheeler Aligner (Li and Durbin, 2009). To ensure the accuracy of the SNP detection, duplicates were removed using the Picard Mark Duplicates tool^1^. The local realignment and SNP detection were performed using the Genome Analysis Toolkit (GATK) protocol^2^ (McKenna et al., 2010) according to the position of the clean reads on the reference genome.

### Genotyping and Effective Marker Detection

The SNPs that were polymorphic between the two parents were selected and classified into eight segregation patterns (ab × cd, ef × eg, hk × hk, lm × ll, nn × np, aa × bb, ab × cc, and cc × ab). Only SNPs with the aa × bb pattern were selected for further analysis. The SNPs with sequencing depth less than 4 were removed. To find recombination breakpoints, we used a sliding window that contained 15 SNPs to scan the genome, one SNP per slide. The window was typed as aa when the number of aa-type or bb-type in the window was >11; in all other cases, the window was typed as ab. After genotyping, the SNPs without recombination were integrated into one bin marker. Markers that had an unusual appearance, low coverage, and/or obvious segregation distortions were filtered prior to linkage analysis. Only the bin markers longer than 10 Kb were reserved for analysis, and bin markers with abnormal base and segregation distortion (*P* < 0.01) were removed.

### Genetic Linkage Map Construction

The selected bin markers were used to construct the genetic linkage map. The bin markers were divided in linkage groups (LGs) based on their locations on the mung bean reference genome; that is, 11 LGs were preliminary set before mapping. For each LG, the mapping data were formatted according to the instruction of Ici-Mapping. The marker order was inferred nnTwoOpt, in which nearest neighbor and two-opt were used for tour construction and improvement, respectively, and recombinant frequencies were calculated with a minimum logarithm of odd (LOD) threshold of 3. Genetic distances were estimated with the Kosambi’s mapping function. Rippling was performed using SARF (sum of adjacent recombination frequencies) with a window size of 5 (Meng et al., 2015).

### Observation and Measurement of Leaf Blade Characteristics

To obtain precise locations of the shapes in the mung bean genome, both the parents and the RILs were investigated for their leaflet shapes under two continually cropping seasons. In the summer of 2018, the RILs were planted in the test site of the Institute of Crop Sciences at the Chinese Academy of Agricultural Sciences (CAAS) (39.96°N and 116.33°E). After harvesting, they were planted in Yazhou (18.38°N and 109.19°E) in winter of 2018 and then in Shunyi (40.23°N and 116.56°E) in summer of 2019. During the flowering stage, a top leaflet of classical ternate compound leaf was collected from the middle part of each plant photographed. For each line, the top leaflets from five plants were evaluated. Because both the lobed and indented leaflets are not classical qualitative traits, to avoid the statistical error in evaluation by visual observation, for the QTL analysis, we counted the number of apexes around each leaf margin for the indented shape, and applied two indexes to evaluate the degree of the lobed leaflet. One index was the ratio of leaf areas, which was calculated using a self-written protocol in Matlab as *R* = (V/A) × 100, where V is the actual area of the leaflet and A is the sum of the actual and lobed areas. The other index was the ratio of the length and width, which was calculated as *Q* = (L_1_/L_2_) × 100, where L_1_ is the distance between the intersection of the concave point on both sides of the leaf and the main vein intersection to the base of the leaf, and L_2_ is the distance from the tip to the base of the leaf. Lines with leaflets that showed both lobed and indented characters were removed from the QTL analysis because, when photographed, the curled margins made them difficult to evaluate.

### Analysis of QTL and Candidate Genes

Quantitative trait locus analysis was conducted using the inclusive composite interval mapping (ICIM) method implemented in IciMapping (Meng et al., 2015). *R* and *Q* values were used to locate QTL for lobed leaflet and the number of apexes (NA) was used to locate QTL for indented leaflet. The ICIM was performed every 0.1 cM with the probability in stepwise regression set as 0.001. For the LOD threshold LOD = log_10_ (P_1_/P_2_), P_1_ is the probability that the site has a QTL and P_2_ is the probability that the site has no QTL. Each QTL was determined by a 1000 permutation test at *P* = 0.05.

The bin markers were located in the mung bean reference genome using the re-sequencing data. According to the physical location of a bin marker on each side of a QTL, the target region in the reference genome was searched on NCBI website^3^. The genes in the target regions were obtained from the GFF3 file and the KOBAS 3.0 database^4^ was used for the gene ontology (GO) and KEGG (Kyoto Encyclopedia of Genes and Genomes) analysis by inputting the gene list and selecting the “Gene-list enrichment” module. The KEGG pathway was considered to be significantly enriched when the corrected *P*-value was <0.05.

## Results

### Sequencing and Genotyping

Totally, 10.99 Gb and 13.35 Gb of clean bases for parents *Dahuaye* and *Jilyu9-1*, respectively, and 494.03 Gb for offspring were obtained. The average Q30 for parents and offspring were 92.86% and 92.19%, and the average GC contents were 34.30% and 35.18%, respectively. The ratios of mapped clean reads for parents were 95.90% (*Dahuaye*) and 95.71% (*Jilyu9-1*), and 94.05% for offspring (Supplementary Table S1).

A total of 564,449 SNP was identified between the parents, and 485,977 (*Dahuaye*) and 485084 (*Jilyu9-1*) of them were homozygous. However, only 23,170 of these SNPs had the aa × bb segregation pattern. These SNPs were further combined into 2202 bin markers for map construction.

### Construction of the Genetic Linkage Map

**FIGURE 1:**
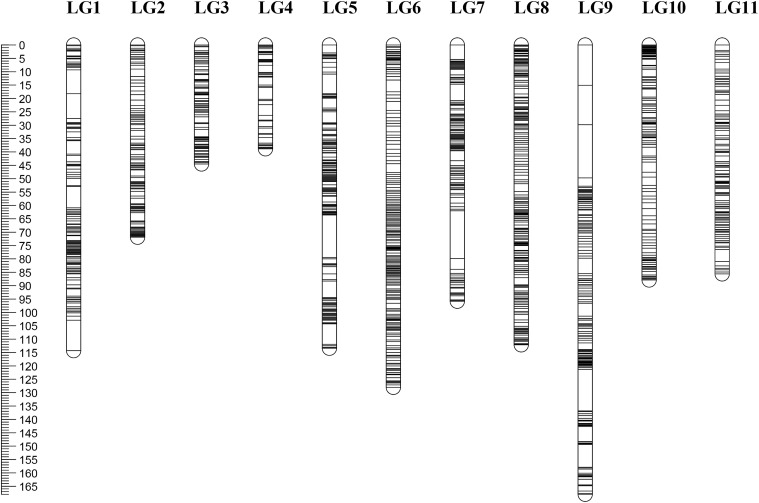
High-density genetic map of *Vigna radiata*. Map distances based on the Kosambi map unit (cM) are provided on the left.

Among the 190 RIL lines, 15 lines had a fairly high ratio of abnormal SNPs (>2% of the total number of SNPs). Therefore, only 175 lines were used for map construction. After linkage analysis, a total of 1946 bin markers consisting of 21,508 SNPs were finally located onto the 11 LGs. According to the marker positions, there was a good agreement between the LGs and the chromosomes of the mung bean reference genome (Supplementary Table S2). The total length of the new map was 1060.17 cM with an average distance between adjacent markers at 0.54 cM. The number of mapped markers in each LG ranged from 99 (LG4) to 258 (LG5) with an average of 177. The average length of the 11 LGs was 96.38 cM, LG4 was the shortest (38.76 cM), and LG9 was the longest (168.03 cM). The density of markers on each LG varied greatly with LG2 (0.35 cM) > LG4 (0.40 cM) > LG5 (0.44 cM) > LG3 (0.45 cM) > LG7 (0.48 cM) > LG8 (0.50 cM) > LG1 (0.51 cM) > LG6 (0.56 cM) > LG10 (0.73 cM) > LG11 (0.74 cM) > LG9 (1.02 cM). A total of 18 gaps (>5 cM) were located in LG1, 5, 7, and 9. Seven of the gaps were l > 10 cM; four were on LG9 including the longest one (19.9 cM) (Table 1 and Figure 1).

**TABLE 1:** Information of the novel constructed genetic linkage map for mung bean.

LG	Num. of bin marker	Total distance (cM)	Average distance (cM)	Max gap (cM)	Num. of gaps >5 cM
LG1	223	114.27	0.51	11.4	4
LG2	209	71.90	0.35	3.2	0
LG3	101	44.55	0.45	2.6	0
LG4	99	38.76	0.4	4.4	0
LG5	258	113.37	0.44	15.8	4
LG6	229	127.99	0.56	4.4	0
LG7	202	95.76	0.48	17.9	3
LG8	223	112.09	0.5	2.9	0
LG9	165	168.03	1.02	19.9	7
LG10	121	87.85	0.73	3.8	0
LG11	116	85.60	0.74	4.5	0
Total	1946	1060.17	0.54	−	18

### Inheritance of Different Leaflet Shapes

**FIGURE 2:**
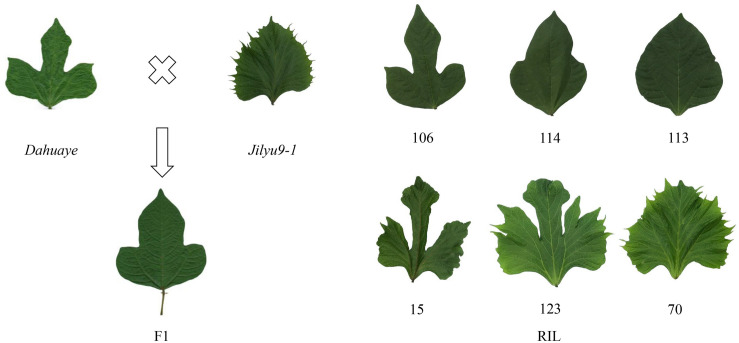
Leaflet shapes of *Dahuaye* and *Jilyu9-1* and their offspring.

The leaflets of all F_1_ plants were semi-lobed, and the indented shape did not appear at the first generation (Figure 2), indicating that the lobed leaflet was semi-dominant and the indented shape recessive. Among their offspring, there were individuals that expressed indented and lobed shapes simultaneously (Figure 2), suggesting these two leaflets were not allelic and were inherited independently.

**FIGURE 3:**
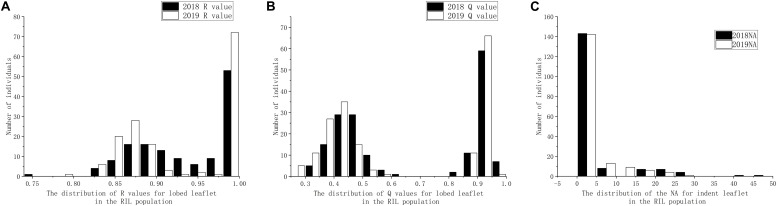
Distribution of leaflet types in the recombinant inbred line (RIL) population derived from *Dahuaye* × *Jilyu 9-1*. **(A)** Distribution of number of apexes (NA). **(B)** Distribution of the length ratio (*Q*) values under different environments. **(C)** Distribution of the area ratio (*R*) values under different environments.

Based on visual observation of the phenotypes, the separation ratio of indented leaflet to ovate shape did not fit 1:1, whereas the separation ratio of lobed leaflet to ovate shape fit 1:1 by both R (χ^2^ = 0.0548, *P* = 0.8149) and Q (χ^2^ = 1.39, *P* = 0.2386) methods. Using the precise methods, both the frequency of distribution of both the indented leaflet by counting the number of apexes and the lobed shape using R and Q methods did not accord with classical normal distribution, under the 2018 and 2019 environments (Figure 3).

### Identification of QTL for the Lobed and Indented Leaflet Traits

**FIGURE 4:**
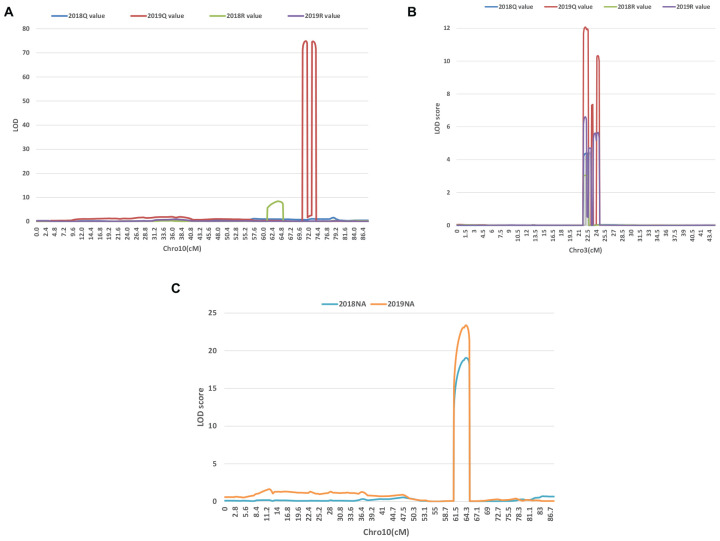
LOD graphs of the QTLs identified for the percentage of lobed leaflet and indented leaflet in a mung bean RIL population of *Dahuaye* × *Jilyu 9-1* in 2 years (2018 and 2019). **(A,B)** LOD plots for lobed leaflet. **(C)** LOD plot for indented leaflet.

**FIGURE 5:**
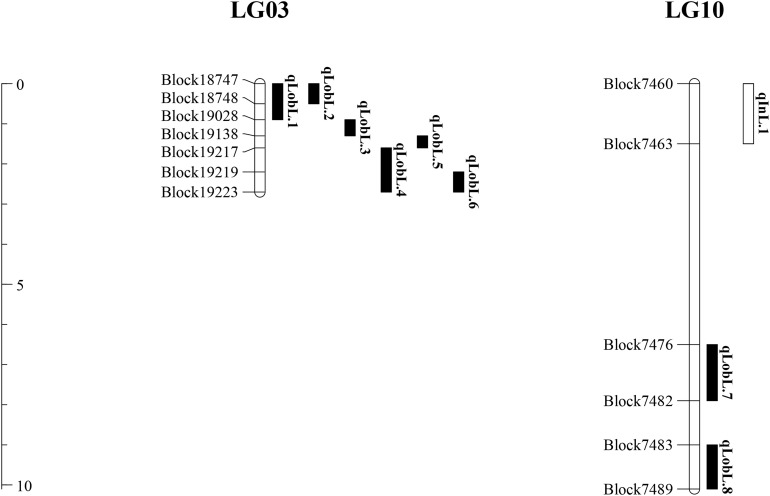
Distribution of QTLs for indented and lobed leaflets in mung bean.

A series of tightly linked peaks on LG3 and LG10 were detected on the LOD contours for the lobed leaflet under the different environments (Figure 4), corresponding with six QTLs on LG3 and two on LG10 (Table 2 and Figure 5). Among them, one major QTL, Block18747–Block 19028 (*qLobL.1*) spanning 1.0 cM on LG3, was stably detected in the 2018 libraries with *Q* and *R* values, with phenotypic variation explained (PVE) values from 50.0 to 69.5%. This stable QTL was physically mapped onto chromosome 3 from 5471015 to 5544796 bp. The other five QTLs on LG3 were identified in the 2019 libraries using *Q* and *R* values, with PVE values from 15.8 to 39.8%. These five QTLs were almost connected together from Block19028 to Block19223, with two overlapped confidence intervals from Block18747 to Block18748 (*qLobL.2*) and Block19219 to Block19223 (*qLobL.4*). Therefore, one confidence interval from Block18747 to Block18748 (*qLobL.2*) was the only repeated QTL that could be detected under the two different environments. Two other QTLs for lobed leaflet from Block7476 to Block7482 (*qLobL.7*) and Block7483 to Block7489 (*qLobL.8*) on LG10 were detected only in the 2019 libraries, with PVE values of 12.2% and 15.5%1, respectively.

**TABLE 2:** Description of QTLs associated with the indented and lobed leaflet in mung bean.

Trait	Method	Environment	QTL name	Chro.	Left marker	Right marker	Physical position	Lod	PVE (%)
Indented leaflet	NA	2018	*qInL.1*	10	Block7460	Block7463	16431849–16788088	19.1	39.7
		2019	*qInL.1*	10	Block7460	Block7463	16431849–16788088	23.4	45.5
Lobed leaflet	*R* value	2018	*qLobL.1*	3	Block18747	Block19028	5471015–5941848	6.1	50.0
		2019	*qLobL.2*	3	Block18747	Block18748	5471015–5544796	66.1	27.7
			*qLobL.3*	3	Block19028	Block19138	5941848–6074518	47.0	16.7
			*qLobL.4*	3	Block19217	Block19223	6181985–6235477	56.2	19.5
	*Q* value	2018	*qLobL.1*	3	Block18747	Block19028	5471015–5941848	8.8	69.5
		2019	*qLobL.2*	3	Block18747	Block18748	5471015–5544796	120.7	29.1
			*qLobL.5*	3	Block19138	Block19217	6074518–6181985	73.7	15.8
			*qLobL.6*	3	Block19219	Block19223	6214542–6235477	103.3	17.4
			*qLobL.7*	10	Block7476	Block7482	17381168–17531051	74.9	12.2
			*qLobL.8*	10	Block7483	Block7489	17619040–17858382	74.8	14.5

*NA, the number of apexes around each leaf margin for the indented shape; PVE, phenotypic variation explained value for a QTL.*

For indented leaflet, one common QTL (*qInL.1*) on LG10 was detected in the 2018 and 2019 libraries. It was located from Block7460 and Block7463 and spanned about 2.4 cM, with PVE values of 39.7% (2018) and 45.5% (2019), respectively. QTL *qInL.*1 was tightly linked with *qLobL.7* on LG10.

### Candidate Gene Annotation

We detected 112 genes in all QTL regions related with lobed leaflet, however, only 10 of them were located in the common Block18747–Block18748 region in LG3. Seven of the 10 genes had only non-synonymous SNPs between the parents (Table 3). The products of the homologous genes included polyadenylate-binding protein, late embryogenesis abundant protein, and different protein kinases. The KEGG analysis indicated that *LOC106757470* and *LOC106757471* were involved in four pathways, namely RNA degradation, ubiquitin-mediated proteolysis, RNA transport, and mRNA surveillance, and that *LOC1065757470* was involved in three of these pathways; the exception was ubiquitin-mediated proteolysis.

**TABLE 3:** SNPs between parents in genes related with lobed leaflet in mung bean.

Gene name	Total SNPs	SNPs in exon	Non-synonymous SNPs
LOC106757470	32	14	11
LOC106757280	11	7	5
LOC106757335	10	4	3
LOC106757671	51	8	7
LOC106757416	12	4	2
LOC106757422	3	3	1
LOC106757412	14	4	2

We detected 45 genes located in the QTL region related with the indented leaflet, and 35 of them encoded homologous products. However, only five had SNPs between the two parents. Four SNPS were in the non-coding regions of *LOC106774574*, *LOC106776045*, *LOC106774382*, and *LOC106776095*, and seven SNPs were synonymous mutations in *LOC106775793*. However, the only one SNP could cause an amino acid change, namely tryptophan (*Dahuaye*) to a stop codon (*Jilyu 9-1*).

## Discussion

Several maps have been constructed for mung bean with different marker types: RFLP (Young et al., 1992; Lambrides et al., 2000; Humphry et al., 2002), AFLP (Chaitieng et al., 2002), and SSR (Kajonphol et al., 2012; Sompong et al., 2012; Liu et al., 2016; Wang et al., 2016; Singh et al., 2018). The frequent development of SSR had made them popular both in map construction and gene tagging for more than a decade (Han et al., 2005; Wang et al., 2016). However, the densities of these maps were low because of the low level of variation of SSR markers (Wang et al., 2009). SNPs have gradually become more widely used for high density map construction, including in species related to mung bean (Liu et al., 2016; Pan et al., 2017; Amkul et al., 2019; Somta et al., 2019). With the release of the whole genome of mung bean (Kang et al., 2014), two SNP maps were reported (Schafleitner et al., 2016; Mathivathana et al., 2019). However, because SNP map information is not easily shared among different labs, we constructed our own SNP map.

The order of the markers on LGs in our map agrees well with their locations on the reference genome of mung bean. We consider that the newly constructed map is an improvement over the map constructed by Schafleitner et al. (2016), because in their map, LG3 and LG4 were merged together. The other previous map also agreed well with the reference genome (Mathivathana et al., 2019); however, our map has higher density (0.54 cM) than theirs (3.23 cM), mainly because of the different mapping populations and sequencing method.

The total length of our map was much shorter (1060.2 cM) than that of the previous map (1291.7 cM), and the length of the LGs for the two maps also was quite different. For example, in our map, LG9 was the longest (168.03 cM) and LG2 was the shortest (71.9 cM), while in the previous map, LG7 was the longest (149.1 cM) and LG3 the shortest (90.2 cM), whereas in the previous map, LG7 was the longest (149.1 cM) and LG3 the shortest (90.2 cM). In our map, LG9 was the lowest (1.02 cM), followed by LG11 (0.74 cM) and LG10 (0.73 cM), whereas in the previous map, LG8 had the lowest density (3.03 cM), followed by LG10 (3.02 cM) and LG9 (2.91 cM). However, the total trends of the two maps were similar, suggesting that the two maps were in a good agreement. Although the average interval distance was good enough for precise mapping, there were 18 large gaps in our map, especially on LG9, leading to the low density of this LG. Such gaps are seldom found in maps using traditional markers for several main reasons, including that we used a map population derived from two cultivated genotypes, and the genetic backgrounds were more similar than that between a wild and a cultivated genotype. Inaccuracies in the reference genome may be another reason for the gaps in our map.

To validate the usefulness of the present map, we investigated QTL related with two types of leaflet shapes, lobed and indented. Lobed leaves are common in plants (Zhang et al., 2018), and they also have been reported in mung bean (Jiao et al., 2016). The phenotypes of F1 and F2 plants indicated that the lobed leaflet was semi-dominant and was controlled by multiple genes, and this finding was validated by the QTL analysis. Several QTLs that were tightly linked together were detected on LG3 and LG10. However, only one was consistently found under different environments both with Q and R methods. This QTL spanned from *Block18747* and *Block 18748* on LG3, with PVE from 27.7 to 69.5%. The location of this QTL was not overlapped with the other QTLs on our map but was tightly linked with a QTL predicted by Jiao et al. (2016). Whether these two QTL have different allelic genes needs to be further validated. The other QTLs were only found in 2019 library, including two on LG10, indicating these QTLs were easily affected by the environments.

Ten genes were located in the target region of the common QTL related with lobed leaflet and all 10 genes had SNPs between the parents; however, non-synonymous SNPs were found only in seven of these genes. The metabolic pathways involving these genes were mostly unknown, so it was difficult to identify candidate gene associated the development of lobed leaflet. However, overexpression of the gene encoding a glycosyltransferase has been reported to affect the leaf veins in tobacco (Ma et al., 2012), and this gene has been predicted to be homologous with *LOC106757416*. Therefore, we consider this gene is the most probable candidate gene associated with the lobed leaflet trait.

The indented leaflet is a rare mutant that we found recently in the test field; it has not been reported previously. The indented shape may be caused by the visually observed abnormal variation at the shoot apical meristem, and it also showed differences in the number of apexes around the leaflet margins. This trait was considered recessive because it was absent in F1 plants and, based on our observation of F2 plants, it may be controlled by multiple genes. However, we detected only one major QTL spanning Block7460–Block7463 on LG10, and explaining 39.7% and 45.5% of PVE under the 2018 and 2019 environments. Although 35 genes with homologous annotations were found within the target region of the major QTL related with indented leaflet, only five genes had 11 SNPs between the parents. Among these SNPs, some were located in non-coding regions and some led to synonymous mutations, only one SNP in the coding region of *LOC106775793*, resulted in a synonymous mutation. In addition, *LOC106775793* was homologous with the gene encoding transcription factor TCP2, which is expressed mainly in rapidly growing tissues or organs and may be related with the development of plant cells (Palatnik et al., 2003; Koyama et al., 2017; Bresso et al., 2018). Overexpression of *TCP* reduced the degree of indent leaflet (Koyama et al., 2017), and knocked out of *TCP* led to shrinkage of the leaf margin (Koyama et al., 2010). Therefore, *LOC106775793* may be the most probable candidate gene controlling the indented leaflet trait. The QTL analysis not only validated the novel map as useful for fine mapping of other traits but also provides a base for investigating the molecular mechanism controlling leaflet shapes in mung bean and other legumes.

## Conclusion

We constructed a high-density genetic linkage map for mung bean using re-sequencing technology, and the map agreed with the reference genome of mung bean. QTLs related with different leaflet shapes were analyzed using this map. One major QTL for indented leaflet was detected on LG10 and several tightly linked QTLs on LG3 and LG10 related with lobed leaflet were detected, but only one of them was consistently detected under different environments with different methods. Genes involved in the molecular mechanism of leaf shape also were predicted. The present map will be useful for fine mapping of genes, and the QTLs and candidate genes will shed light on the development of leaflet shapes in mung bean and other legumes.

## Data Availability Statement

We uploaded the raw data of the re-sequencing to NCBI: PRJNA656124.

## Author Contributions

LW conceptualized, designed, supervised the study, and revised the manuscript. JW and JL developed the population and data analysis. ZL, XY, HC, SW, and XiC conducted the data collection. LW and XuC secured the research funding. JW wrote the manuscript. All authors read and approved the final manuscript.

## Conflict of Interest

The authors declare that the research was conducted in the absence of any commercial or financial relationships that could be construed as a potential conflict of interest.

## Footnotes

1 http://sourceforge.net/projects/picard/

2 https://www.broadinstitute.org/gatk/guide/best-practices?bpm=DNAseq#variant-discovery-ovw

3 https://www.ncbi.nlm.nih.gov/genome/?term=mungbean

4 http://kobas.cbi.pku.edu.cn/anno_iden.php
